# Comparison of the Therapeutic Effects of Acupuncture at PC6 and ST36 for Chronic Myocardial Ischemia

**DOI:** 10.1155/2017/7358059

**Published:** 2017-08-16

**Authors:** Li Shi, Jiliang Fang, Jiping Zhao, Guiyong Liu, Qing Zhao, Jinling Zhang, Jinzhao Zhang, Bing Zhu, Fanrong Liang, Peijing Rong

**Affiliations:** ^1^Dongzhimen Hospital Affiliated to Beijing University of Chinese Medicine, Beijing 100700, China; ^2^Institute of Acupuncture and Moxibustion, China Academy of Chinese Medical Sciences, Beijing 100700, China; ^3^Guang'anmen Hospital, China Academy of Chinese Medical Sciences, Beijing 100053, China; ^4^Chengdu University of TCM, Chengdu 610075, China

## Abstract

We aimed to compare the differences of the effects on chronic myocardial ischemia (MI) of acupuncture at PC6 and ST36. The chronic MI model of minipigs was created by implanting an Ameroid constrictor on the left anterior descending coronary artery (LAD) and then two weeks' acupuncture was stimulated at PC6 or ST36, respectively. The results showed that both acupoints' stimulation decreased the serous cardiac troponin T (cTnT) and ischemia modified albumin (IMA) significantly and improved the ischemic ECG changes. The amplitude of pathological Q wave in the PC6 group decreased more significantly than that of the ST36 group. The cardiovascular magnetic resonance imaging (cMRI) results showed that the decreased left ventricular ejection fraction (LVEF) was not improved obviously in both groups. The left ventricular end-diastolic volume (LVEDV) and left ventricular end-systolic volume (LVESV) enlarged progressively even after acupuncture. The left ventricular wall mass (LVWM) in the ST36 group increased more obviously than that of the PC6 group, which paralleled the decreasing angiotensin II (Ang II) concentration in the plasma. These results suggested that acupuncture at PC6 or ST36 was effective for protecting the myocardium from chronic ischemic injury, and the effect of PC6 seemed to be better.

## 1. Introduction

Ischemic heart disease (IHD), which threatens human life and health seriously, is the primary cause of disability-adjusted life years (DALYs) worldwide [1]. Coronary atherosclerosis leads to chronic MI, which leads to myocardium adaptive and compensatory remodeling. It manifests myocyte hypertrophy and increased interstitial fibrosis. It brings about chronic and progressive myocardial contractile and relaxant dysfunction. Medications and revascularization therapy are in common use and effective for acute myocardial infarction, unstable angina, or chronic stable angina. But the efficacy is not as ideal as expected so far. Medications may result in some adverse side effects, and recurrent angina after revascularization may occur because of restenosis, revascularization failure, and the progression of atherosclerosis [2, 3].

Acupuncture showed beneficial effects on IHD without the obvious side effects. In the latest decades, the clinical researches have revealed that acupuncture can play an important role as a complementary therapy to relieve angina on the basis of drug administration [4–6]. Recently, a randomized clinical trial was conducted in multicentres with two-year follow-up. In the study, electroacupuncture pretreatment (EAP) was performed 1-2 h prior to the percutaneous coronary intervention (PCI). The results indicated that EAP could reduce the incidence of myocardial infarction of type 4a in 24 h after PCI, and decrease the major adverse cardiac/cerebrovascular event (MACCE) rate in 2 years after PCI [7].

PC6 is the major acupoint to be used for treating IHD since ancient times according to the acupuncture classical theory. It is used most frequently in treating angina according to the results of a data mining analysis [8]. The animal experiments indicated that the mechanism of acupuncture at PC6 on IHD acted on multilevels and multitargets. It can affect the activity of autonomic nervous nucleus in the centre [9–12], inhibit the sympathoexcitation [13], and decrease the cardiac norepinephrine release [14] to lower oxygen demand. It can also modulate the response of target organ, for example, affecting the expression of gene in the cardiomyocytes [15] and the activity of signal transduction pathway [16, 17]. Acupuncture at ST36 induces a tonic effect for the body according to acupuncture theory and clinical practice, which is also beneficial for the MI. Evidences have showed that acupuncture at ST36 could increase the excitement of vagal nerve [18, 19], inhibit the cardiac sympathetic center, and decrease the sympathetic outflow [20].

Acupuncture at PC6 and ST36 is often used together as a complementary treatment to treat the conditions of MI and heart failure. However, there is no clinical trial or animal study to clarify the difference of the efficacy of both acupoints. Our current study was conducted to compare the effects between PC6 and ST36 on the minipig model with chronic MI, and the objective indications were measured by ECG, serous biomarkers, and the left ventricular heart function by cMRI.

## 2. Material and Methods

### 2.1. Material and Design

Twelve Chinese minipigs were recruited (8-9 months old, 28.22 ± 4.73 kg). All the minipigs were supplied by the Animal Experimental Centre of Beijing Tongheshengtai Comparative Medicine Institute (permission number of SCXK (Jing) 2015-004). The breeding and experimental treatment of minipigs were carried out in accordance with the national guidelines for the use of experimental animals. All the experimental protocols were approved by the Animal Welfare and Ethics Committee in Institute of Acupuncture and Moxibustion, China Academy of Chinese Medical Sciences (permission number of 20130013).

**Figure 1:**
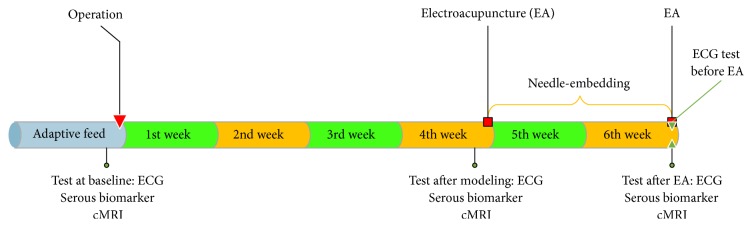
The experimental procedure.

The animals were kept separately at constant temperature of 18–22°C. After the minipigs had been fed for 7 days to adapt the environment, a series of tests were performed at the normal basic condition, including ECG, venous blood sample, and cMRI. Venous blood sample was taken for testing the serous creatine kinase-MB (CK-MB), IMA, cTnT, and Ang II. All the minipigs were separated into two groups randomly, PC6 (*n* = 6) and ST36 (*n* = 6) groups. The minipigs were operated for implanting Ameroid constrictor on the anterior descending coronary artery to create the chronic MI model. Four weeks after the operation, the tests mentioned above were conducted again. At the time of the 4th week, a long-effect acupuncture stimulation technique, which is named as the needle-embedding in the subcutaneous tissue of the acupoint (NE), was performed for 2 weeks. Electroacupuncture (EA) was stimulated twice at the 4th and 6th week for all minipigs too. After NE and EA intervention, all the tests above were conducted to assay the therapeutic effect (Figure 1).

### 2.2. Operation

All the minipigs were fasting and free of water for 24 hrs before the operation, and the anesthesia was conducted by Pentobarbital sodium (Merck, Germany) (15 mg/kg, im) combined with Sumianxin II (Jilin Huamu Animal Health Products Co., Ltd., China) (0.1 ml/kg, im) or an equivalence mixture of Ketamine (Fujian Gutian Pharmaceutical Co., Ltd., Fujian, China) and Sumianxin II (0.28 ml/kg, im). Catheter tee was placed into the marginal ear vein. Endotracheal tube was inserted for assisting respiration by respirator. Multiparameter monitor (type G3H) (JieNarui Medical Instrument Co., Ltd., Shenzhen, China) was connected. The minipigs were lied on the right side, and an incision was operated on the left side of the third intercostal space. The pericardium was cut off at the left atrial appendage and the LAD could be showed out; then about 1.5 cm LAD was separated out from the epicardium and adipose tissue carefully. The Ameroid constrictor was implanted on the proximal-middle section of the LAD by sliding the inner side ring to stagger the gap of inner and outside ring. The inner diameter of Ameroid constrictor is 2.5 mm. The outside ring is stainless steel, and the inner side ring is casein, which can continuously swell inwards when absorbing moisture. In this way the LAD can be restricted and the chronic MI was produced. The pericardium was sutured after making sure there was no active hemorrhage. When autonomous respiration was recovered, the tube was drawn out.

### 2.3. Electroacupuncture and Needle-Embedding

The acupoint position of PC6 and ST36 on the minipigs was confirmed by combining the point selection principle used by human and comparative anatomy. The position of PC6 is on the connecting line of the ulna olecranon nodules and the concave of the radial remote dorsal articular surface. The acupoint locates one-sixth of the line away from the remote side of the radius, which is between the ulna and radius on the palm side [21]. The position of ST36 is at the depression below and anterior capitula fibula, which is outside and below the knee. The sterile acupuncture needles (0.4 × 40 mm, Huatuo brand, Suzhou Medical Supplies Co., Ltd., China) were pricked into the bilateral PC6 or ST36 and connected with acupoint and nerve stimulator (HANS-200A, Hanshi brand, Nanjing Jisheng Medical Technology Co., Ltd., China). EA was performed with 2 Hz frequency and 6 mA intensity and lasted for 20 mins each time. NE lasted for 2 weeks. Disposable wire-embedding needle was pierced at the acupoints. The tongue was drawn out, and a sterile steel needle (0.3 mm × 1.5 cm) was placed under the subcutaneous along the pinhole, which was fixed with medical proof fabric and bound up.

### 2.4. ECG

ECG was recorded by the 12-lead resting electrocardiograph (ELI350, Mortara Instrument, Inc., America) four times. The minipigs were located at supine position. Three standard bipolar limb leads and the chest leads were recorded. The electrodes were placed as previously described [22]. The speed of the paper was 25 mm/s, and the sensitivity was 10 mm/mV.

### 2.5. Cardiovascular Magnetic Resonance Imaging

The minipigs were kept anesthetic as described above. The MAGNETOM Skyra 3T MR Scanner (Siemens, Erlangen, Germany) was used to scan the cardio-MRI for assaying the left ventricular function, including ejection fraction, left ventricular end-systolic and diastolic volume, stroke volume, cardiac output, and wall mass. The MRI special nonmagnet ECG electrodes were placed on the chest. Four electrodes were placed on left medioclavicular line of the second and fifth intercostal space and on the left border of sternum of the second and fifth intercostal space separately.

Fix the position of two-chamber, four-chamber, and short-axis by using Turbo FLASH (TFL) sequence. Under this position, the T1 weight imaging (T1WI) and T2 weight imaging (T2WI) of the turbo spin echo (TSE) sequence were scanned for the anatomical structure of heart. The parameter was TR = 800 ms, TE = 71 ms, FOV = 340 mm × 276 mm, slice thickness = 5 mm, slice distance = 2.5 mm, and FA = 180 degrees. Cine scanning was performed in short axis, two-chamber, and four chamber position, and the parameter was TR = 39.24 ms, TE = 1.43 ms, FOV = 340 mm × 285 mm, FA = 65 degrees, slice thickness = 6 mm, slice distance = 1.2 mm, slice 1, and phase encoding: A-P, segments = 15. The movement of heart and heart function were tested under this sequence.

### 2.6. Blood Sample Taking and Myocardial Ischemia Biomarkers Testing

The minipigs were located at supine position and blood was taken from precaval vein. Serous concentrations of CK-MB and IMA were tested by colorimetry with CK-MB Assay Kit (BioSino, Technology & Science Inc., China) and IMA Assay Kit (Beijing Huaying Institute of Biotechnology, China) and by using Hitachi 7160 automatic biochemical analyzer (Japan Hitachi company, Japan). Serous concentrations of cTnT and Ang II were tested by radioimmunoassay with cTnT and Ang II Assay Kits (Beijing Huaying Institute of Biotechnology, China) and by using r-911 automatic radioimmunoassay (RIA) counter (Beijing University of Science and Technology Industrial Company). The methods and procedures followed the protocols of the test kits after separating serum.

### 2.7. The Methods of Statistics

The measurement data were represented by mean ± standard error of the mean (mean ± SEM). The comparison between two groups adopted *t*-test or rank sum test. The enumeration data were represented by rate. The comparison of ratio adopted four plate chi-square test or *R* × *C* Fisher exact method. It is considered statistically significant when the *P* value is less than 0.05. The statistical analysis was conducted by the IBM SPSS 20.0 software (IBM China Company).

## 3. Results

Initially, 12 minipigs were recruited in the study. One minipig of the ST36 group died in the operation and the cMRI data of two in PC6 groups were failed to collect completely. The data of ECG, the serous biomarkers (PC6 group, *n* = 6, and ST36 group, *n* = 5), and cMRI (PC6 group, *n* = 4; and ST36 group, *n* = 5) were analyzed.

### 3.1. ECG

**Figure 2:**
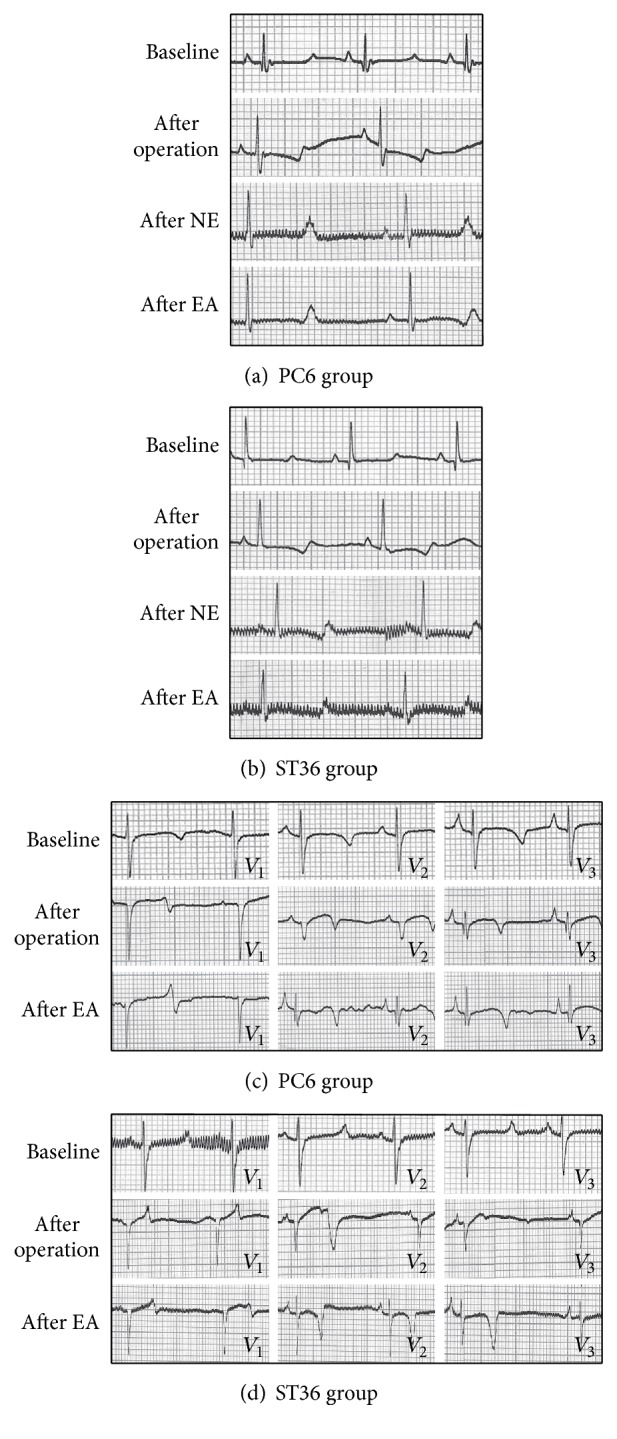
Examples of lead II and leads* V*_1_–*V*_3_ ECG of the PC6 and ST36 groups minipigs before (baseline) and after the operation and after needle-embedding (NE) and electroacupuncture (EA). (a)-(b) Examples of lead II ECG of the PC6 and ST36 groups before (baseline) and after operation, after NE, and after EA. It showed that the ST segment declined and T wave became bidirectional after operation. After acupuncture, ST segment came back to basic line, T wave became upward, and the QT interval prolonged significantly after acupuncture at PC6 and ST36. (c)-(d) Examples of leads* V*_1_–*V*_3_ ECG of the PC6 and ST36 groups before (baseline) and after operation and after EA. It showed that the pathologic Q wave appeared at lead* V*_1_–*V*_3_ at the 4th week after the operation, and the pathologic Q wave disappeared at leads* V*_2_ and* V*_3_ after acupuncture at PC6 and ST36.

**Figure 3:**
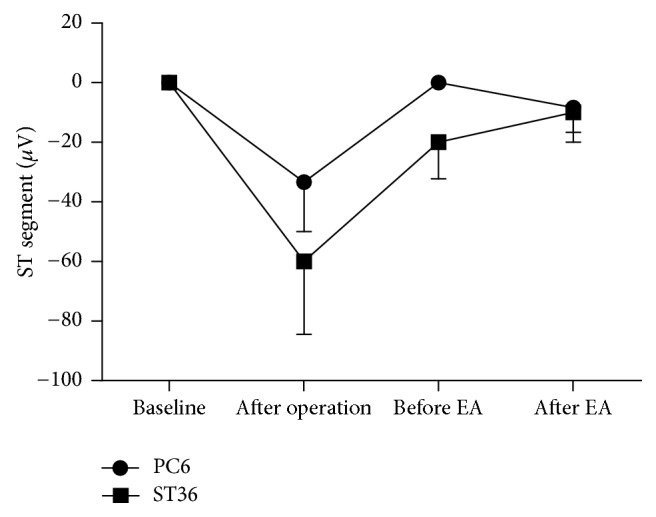
The variation tendency of the ST segment amplitude of lead II. The ECG was taken at the normal condition, at the 4th week after operation, and at the 6th week after operation (before and after EA). The declined ST segment amplitude decreased after acupuncture both in the PC6 group (*n* = 6) and in the ST36 group (*n* = 5). But there were no significant statistical differences. Data are expressed as mean ± SEM. Statistical analysis was performed by Wilcoxon signed-ranks and Mann-Whitney.

**Figure 4:**
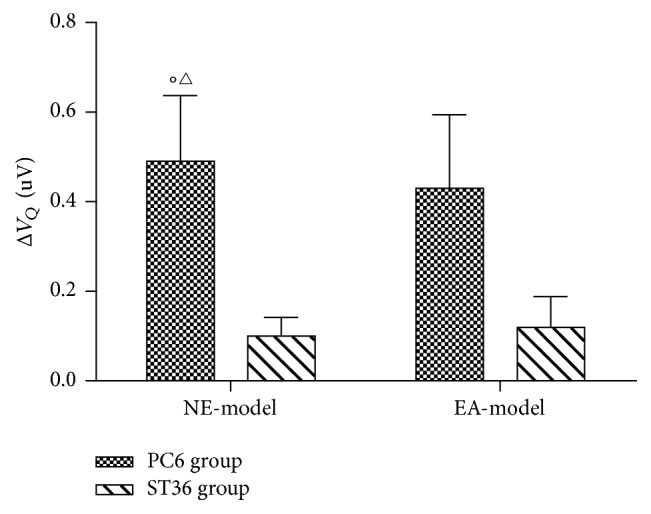
The changed amplitude of pathological Q wave. The figure shows the changes in pathological Q wave amplitude of lead* V*_1_, which is the differences of pathological Q wave amplitude after needle-embedding (NE) or electroacupuncture (EA) and after the operation. The amplitude of pathological Q wave decreased significantly after NE at PC6 acupoint (^○^*P* = 0.029, compared with 0 mV), and there was statistical difference between the PC6 and ST36 group (^△^*P* = 0.034, compared with that of the ST36 group). Data are expressed as mean ± SEM and were analyzed by *t*-test. *N* = 5 in both PC6 group and ST36 group. One minipig in the PC6 group did not record complete ECG.

The ECG showed the sign of MI at the 4th week after the operation. The ST segment in lead II of both groups declined horizontally or slantly, but there was no statistical significance compared with the basic amplitude of ST segment (*P* > 0.05) (Figures 2(a), 2(b), and 3). 83.3% T wave of lead II in the PC6 group and 80% of that in the ST36 group became inverted or bidirectional at the 4th week after operation (Table 1). After NE and EA, the declined ST segment amplitude of the two groups decreased, but there was no statistical significance (*P* > 0.05, compared with that after operation of the same group and between the two groups) (Figures 2(a), 2(b), and 3). A portion of inverted and bidirectional T wave recovered to normal, which occupied 50% in the PC6 group and 40% in the ST36 group, but there was no statistical significance (*P* > 0.05) (Table 1). The pathological Q wave is a symbol of myocardial infarction or myocardial ischemic injury. It appeared at leads* V*_1_–*V*_3_ at the 4th week after the operation, which was consistent with the blood supply area of LAD. After acupuncture, the incidence of pathological Q wave decreased in both groups. It appeared only at lead* V*_1_ in the ST36 group but still appeared at leads* V*_2_ and* V*_3_ in some individuals of the PC6 group (Table 2 and Figures 2(c) and 2(d)). The amplitude of pathological Q wave of the both groups decreased, and there was statistical significance in the PC6 group after NE (*P* = 0.029). There was also statistical significance between the two groups after NE (*P* = 0.034) (Figure 4).

**Figure 5:**
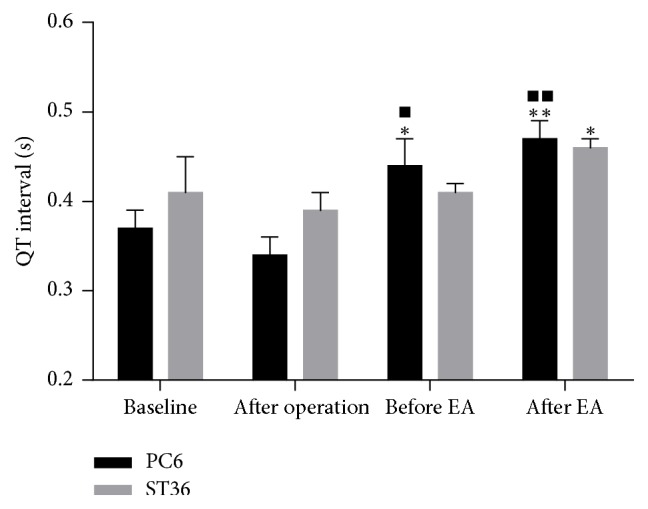
Lead II QT interval of the PC6 group and ST36 group. Data are expressed as mean ± SEM and were analyzed by *t*-test. ^*∗*^*P* < 0.05 and ^*∗∗*^*P* < 0.01, compared with that after operation in the same group. ^■^*P* < 0.05 and ^■■^*P* < 0.01, compared with the baseline of the PC6 group. The QT interval of the PC6 group prolonged significantly compared with that after operation (*P* = 0.021, after NE; and *P* = 0.003, after EA). The QT interval of the ST36 group prolonged significantly after EA compared with that after operation (*P* = 0.024). The QT interval of the PC6 group prolonged significantly compared with the normal condition (*P* = 0.021, after NE; and *P* = 0.004, after EA).

Under the normal condition, the QT interval of PC6 group and ST36 group is (0.37 ± 0.02) s and (0.41 ± 0.04) s, respectively. After operation, the QT interval shortened slightly. After acupuncture at PC6 and ST36, the QT interval prolonged progressively, and there was statistical significance compared with that after operation (The PC6 group: *P* = 0.021 after NE, and *P* = 0.003 after EA; the ST36 group: *P* = 0.024 after EA). It was also found that the QT interval of the PC6 group significantly prolonged compared with the normal condition (*P* = 0.021, after NE; *P* = 0.004, after EA) (Figures 5, 2(a), and 2(b)).

### 3.2. Serous CK-MB, cTnT, IMA, and Ang II

Serous CK-MB, cTnT and IMA are the sensitive and specific biomarkers of myocardial injury. In our study, the serous concentrations of CK-MB and cTnT of both groups increased after operation, but there were no significant differences compared with their separate baselines. The basic level of serous IMA in the ST36 group was significantly higher than that of the PC6 group (*P* = 0.030). Serous concentrations of IMA of the both groups increased after the operation, and that of the PC6 group increased significantly compared with its basic level (*P* = 0.033). Serous concentrations of cTnT and IMA decreased significantly after acupuncture at PC6 and ST36 (*P* = 0.028, cTnT of the PC6 group; *P* = 0.043, cTnT of the ST36 group; *P* = 0.007, IMA of the PC6 group; and *P* = 0.043, IMA of the ST36 group), and the serous CK-MB decreased as well (Table 3).

The changes of serous Ang II between both groups were not of statistical significance at the baseline, the 4th week after the operation, and after two weeks' acupuncture. The serous Ang II of the PC6 group increased at the 4th week after the operation. The decreasing trend of the serous Ang II after acupuncture at PC6 could be seen in our study. The basic serous Ang II of the ST36 group was higher than that of the PC6 group, and there was no significant difference between the two groups. The variation trend of the serous Ang II of the ST 36 group was opposite to that of the PC6 group after the operation and acupuncture (Table 3).

### 3.3. Cardiac Functions

After implanting Ameroid constrictor, coronary artery was stenosed progressively, which led to cardiomyocyte ischemia, infarction, and structure remodeling. The contractile and relaxant function of left ventricle was impaired. The cMRI results showed that the LVEDV and LVESV increased in each group at the 4th week after the operation, but there was no statistical significance compared with their separate baseline. The LVEF of both groups decreased, but only that of the ST36 group had statistical significance as compared with its baseline (*P* = 0.035). After acupuncture at PC6 and ST36, LVEDV, and LVESV increased furthermore, the LVEDV of the ST36 group (*P* = 0.008) and LVESV of the both groups (PC6, *P* = 0.024; ST36 group *P* = 0.043) were significantly larger than their baseline. The LVEF of PC6 and ST36 group was not improved significantly after acupuncture, but the LVEF of ST36 group was increased and still significantly lower than its baseline level (*P* = 0.017). The stroke volume and cardiac output had no significant changes at the time of the 4th week after the operation and after acupuncture. The left ventricular wall mass (LVWM) of the two groups was approximately the same at the normal condition, and it increased progressively. The LVWM of the ST36 group was significantly greater than that of the PC6 group after acupuncture (*P* = 0.030) (Table 4). It was indicated that the cardiac hypertrophy of the ST36 group was more serious than the PC6 group. Acupuncture at PC6 may have better effect on inhibiting cardiomyocyte hypertrophy and myocardial interstitial fibrosis.

## 4. Discussion

The chronic MI model of Chinese minipigs was created by implanting the Ameroid constrictor on the proximal-middle section of the LAD in the present study. The cardiac structure, coronary artery structure, and distribution of the minipig resemble those of the human being [23, 24], and the cardiac ion channel distribution is also similar to that of the human heart [25]. After implanting the Ameroid constrictor, the coronary artery stenosis arises gradually. At the time of the 4th week after the operation, the occlusion of coronary artery accesses 90%–100% [22]. There is only a small part of necrotic myocytes. This model is much more similar to the pathological features of chronic MI patients. This study is the preliminary study to compare the effect of PC6 and ST36 on the chronic MI by using this animal model. So the results of the study are valuable for the acupuncture clinical guidance.

Four weeks after the operation, the ECG showed ST segment declined horizontally or slantly, and T wave inverted or became bidirectional. Pathological Q wave appeared at leads* V*_1_–*V*_3_. The concentration of serous IMA increased significantly. All the above results indicated that the operation created chronic MI model successfully. The amplitude of the pathological Q wave at lead* V*_1_ decreased much more significantly in the PC6 group than in the ST36 group. The incidence of pathological Q wave was decreased in both groups, and it only appeared at lead* V*_1_ in the ST36 group after two weeks' acupuncture stimulation. These phenomena might indicate that acupuncture could reduce the ischemic area. The serous IMA and cTnT reduced significantly after acupuncture at PC6 and ST36. The results of ECG and the serous biomarkers suggested that acupuncture at both PC6 and ST36 has protective effects on the myocardium from ischemic injury, and the effect of the PC6 group was better.

The results of ECG also showed that the QT interval of lead II prolonged significantly after acupuncture at PC6 and ST36. The QT interval reflects the depolarization and repolarization time of the ventricular myocardium. The QRS interval of the minipigs did not change after operation and acupuncture. So the prolonged QT interval revealed that the repolarization time prolonged after acupuncture, which led to prolonged effective refractory period and brought to decreased heart rate. It is beneficial for reducing the myocardial oxygen consumption. It might be the protection mechanism of acupuncture at PC6 and ST36. However, the prolonged QT interval is also caused by heart failure and coronary insufficiency, and it may be closely associated with sudden death [26, 27]. The QT interval is closely related to the RR interval. The abnormal QT prolongation should be determined by the heart rate. The Bazett formula is often used to rectify the QT interval. But the QT interval of porcine lacks a normal standard. So it cannot be confirmed that the prolongation of QT interval is adverse in the present study, and it is necessary to investigate further.

The increased serous concentration of cTnT is a diagnostic “golden standard” for acute myocardial infarction according to ESC/ACC criteria. The diagnostic sensitivity of an elevated IMA for myocardial infarction is 100%, but the specificity is only 34.5%. This is because IMA is a diagnostic index for MI not infarction [28]. In the present study, the serous IMA content was increased significantly in the PC6 group after the operation. The serous concentrations of CK-MB and cTnT in both groups increased without statistical significance at the time of the 4th week after the operation. Combined with the results of the ECG, which lacked the representative manifestation of myocardial infarction, it was indicated that there was no acute myocardial infarction at the time of 4th week after the operation, and remarkable ischemia still existed. Moreover, the serous IMA level at the baseline of the ST36 group was significantly higher than that of the PC6 group, and it was increased after the operation, but there was no statistical significance compared with itself baseline. This result might be due to individual differences and the small sample size. However, the ischemic ST-T changes and pathologic Q wave in the ECG and the cardiac dysfunction shown by cMRI still indicated that there was cardiac ischemic injury in the ST36 group. The serous cTnT and IMA decreased significantly after acupuncture in both groups, which indicated that acupuncture at PC6 or ST36 was able to diminish the myocardial ischemic injury.

The renin-angiotensin system (RAS) excitement is one of the pathological characteristics of IHD. Myocardium and vascular endothelium can express all the elements of the RAS. The RAS can interact with cardiac sympathetic system. Ang II promotes the sympathetic nerve ending releasing norepinephrine, which leads to increasing the myocardiac oxygen consumption. Ang II also plays an important role in ventricular remodeling. It promotes cardiomyocyte hypertrophy and myocardial interstitial fibrosis. In the present study, the serous Ang II of the PC6 group was increased after the operation, which was consistent with previous studies [29, 30]. Cardiac ischemia is able to stimulate the RAS system, and then the serous Ang II concentration increases, while there was a decline trend in the serous content of Ang II in the ST36 group, which was also due to individual differences. The serous Ang II concentration was decreased after acupuncture at PC6 but increased after acupuncture at ST36. It was indicated that acupuncture at PC6 might be beneficial to inhibit the ventricular remodeling induced by chronic myocardial ischemia. It is necessary to explore further to verify the hypothesis.

cMRI is a three-dimensional stereoscopic imaging technique with high precision. It is more sensitive and specific at the aspect of diagnosing cardiovascular disease. The results are not affected by the problem of imaging window and the artificial subjective factors of the operator [31]. cMRI possesses its advantage compared with cardiac ultrasonography. It is a new golden standard for assessing heart function [32]. The present study is the first time to assess the acupuncture effect on heart function by using cMRI. The LVEDV and LVESV increased and the LVEF decreased after the operation, which indicated cardiac dysfunction. It resulted from ventricular remodeling after cardiac infarction [33]. Within weeks and months after myocardial infarction, it is followed by fibroblast proliferation and collagen deposition and scar formation [34]. Histological examination revealed that cell rupture, a reduction in the intercellular space, stretching of myocytes, and slippage of groups of myocytes lead to wall thinning in both infarcted and noninfarcted regions [35]. Ventricular dilation is a compensatory response to maintain stroke volume as the LVEF was decreased [33]. But the dilation would increase the diastolic and systolic wall stress, which would bring about further ventricular enlargement [36]. The results of the present study showed that the LVEDV and LVESV of the both groups increased and the LVEF of the PC6 group decreased further after acupuncture. Although there was an increasing tendency in the LVEF of the ST36 group after acupuncture, it was still significantly lower than that of baseline level. It indicated that acupuncture could not inhibit the pathological progress of cardiac remodeling and improve the cardiac dysfunction. It was also observed that LVWM of the PC6 was significantly less than that of the ST36 group after acupuncture, which indicated that acupuncture at PC6 might be able to inhibit the left ventricular hypertrophy. These results were consistent with that of the serous Ang II concentration. In another study, NE at PC6, BL15, and ST36 for 7 weeks could increase the LVEF significantly in the chronic heart failure rat model [37]. However, the therapy duration was much shorter in the present study. EA or NE at PC6 may inhibit the RAS, decrease serous Ang II, and inhibit the left ventricular remodeling. We speculate that acupuncture at PC6 may have a stronger remote potential effect on the heart systolic function and increase the LVEF.

The present study was an exploratory experiment, and there were some limitations on account of high experimental cost, high morbidity of the operation, the individual variation, and the difficult manipulation on the big animals, as well as the small sample size of the study. Chronic MI leads to ventricular remodeling and heart failure, which is a long-lasting pathologic process. It is reported that the LVEDV is still increased and the LVEF decreased significantly at 3 months after operation in some cases [38]. The chronic MI and heart dysfunction needs long and enduring treatments. Two-week period of treatment was not enough. More indexes are needed in the molecular biology, pathology, high resolution imaging in order to provide more evidences, and mechanisms for acupuncture.

## 5. Conclusions

The results of the present study revealed that both PC6 and ST36 had improvement effect on chronic MI, and PC6 acupoint was more effective for treating this disease. There was no obvious effect on the dilation of left ventricular chamber and systolic dysfunction of left ventricle. But acupuncture at PC6 was seemly capable of restraining left ventricle from hypertrophy, and acupuncture at ST36 had the tendency to improve the LVEF. The results of this study showed the important clinical significance for acupuncture therapy.

## Figures and Tables

**Table 1:** The percentage of normal and abnormal T wave of lead II after the operation and acupuncture.

	After operation		After acupuncture	
	Normal (%)	Abnormal (%)	Normal (%)	Abnormal (%)
PC6 group (*n* = 6)	16.70	83.30	50.00	50.00
ST36 group (*n* = 5)	20.00	80.00	40.00	60.00

It is indicated that a portion of inversed and bidirectional T wave induced by myocardial ischemia come back upward after acupuncture. But there was no statistical significance. Data are represented by rate and were analyzed by four tables' chi-square test.

**Table 2:** The incidence of pathological Q wave in leads *V*_1_, *V*_2_, and *V*_3_.

	PC6 group (*n* = 5)		ST36 group (*n* = 5)	
	After operation	After acupuncture	After operation	After acupuncture
Lead *V*_1_ (%)	42.90	66.70	60.00	100.00
Lead *V*_2_ (%)	42.90	33.30	20.00	0.00
Lead *V*_3_ (%)	14.30	0.00	20.00	0.00

It was shown that the incidence of pathological Q wave decreased at leads *V*_2_ and *V*_3_. But there was no statistical difference. Data are represented by rate and were analyzed by *R* × *C* Fisher exact method. *N* = 5 in both PC6 group and ST36 group. One minipig in the PC6 group did not record complete ECG.

**Table 3:** Serous concentrations of CK-MB, cTnT, IMA, and Ang II of the minipigs in the PC6 and ST36 group tested at the normal condition, at the 4th week after the operation and after acupuncture.

	Baseline		After operation		After acupuncture	
	PC6 group	ST36 group	PC6 group	ST36 group	PC6 group	ST36 group
CK-MB (U/L)	8.39 ± 0.85	9.08 ± 2.92	9.61 ± 1.03	11.16 ± 1.79	7.76 ± 0.90	6.87 ± 0.50
cTnT (pg/ml)	55.43 ± 6.59	59.41 ± 23.09	69.14 ± 7.96	86.12 ± 15.22	40.07 ± 3.87^*∗*^	32.62 ± 0.99^*∗*^
IMA (U/mL)	50.46 ± 7.48	74.11 ± 9.77^△^	76.30 ± 4.11^■^	85.00 ± 7.43	59.07 ± 3.01^*∗∗*^	44.89 ± 1.60^*∗*^
Ang II (pg/ml)	90.94 ± 13.41	134.99 ± 46.39	131.51 ± 20.35	107.82 ± 22.15	119.72 ± 27.62	142.13 ± 16.71

Data are expressed as mean ± SEM (the PC6 group, *n* = 6; the ST36 group, *n* = 5). ^*∗*^*P* < 0.05 and ^*∗∗*^*P* < 0.01, compared with that after operation of the same group. ^■^*P* < 0.05, compared with its baseline of the same group. The data of IMA of the PC6 group at different time were analyzed by *t*-test, and the others were analyzed by Wilcoxon signed-ranks. ^△^*P* < 0.05, compared with the baseline of the PC6 group. Datum comparisons between the two groups used rank sum test followed by Mann-Whitney *U* test.

**Table 4:** The cardiac functional parameters of the PC6 group and ST36 group minipigs.

	Baseline		After operation		After acupuncture	
	PC6 group	ST36 group	PC6 group	ST36 group	PC6 group	ST36 group
LVEF (%)	71.00 ± 6.47	68.60 ± 3.77	54.75 ± 4.50	47.00 ± 4.91^■^	49.00 ± 1.96	51.80 ± 1.62^■^
LVEDV (ml)	43.58 ± 5.26	37.12 ± 4.50	60.22 ± 7.03	56.22 ± 11.74	72.92 ± 6.65	68.92 ± 5.36^■■^
LVESV (ml)	13.26 ± 3.26	10.64 ± 1.17	28.79 ± 5.55	28.82 ± 5.96	37.24 ± 2.63^■^	33.16 ± 3.08^■^
Stroke volume (ml)	29.56 ± 4.31	26.48 ± 3.63	33.94 ± 1.02	26.79 ± 6.26	38.06 ± 4.50	35.76 ± 2.72
Cardiac output (l/min)	1.92 ± 0.42	1.63 ± 0.19	2.17 ± 0.11	2.39 ± 0.10	2.13 ± 0.16	1.76 ± 0.06
LVWM (mean) (g)	34.93 ± 6.81	33.31 ± 4.98	35.65 ± 9.31	37.97 ± 5.21	37.16 ± 2.90	45.88 ± 1.74^△^

The cardiac functions were assessed by cMRI at the normal condition, at the 4th week after the operation, and after acupuncture. Data are expressed as mean ± SEM (the PC6 group, *n* = 4, two in the PC6 group were failed to collect the cMRI data completely; the ST36 group, *n* = 5). ^■^*P* < 0.05 and ^■■^*P* < 0.01, compared with their separate baselines of the same group, respectively (*P* = 0.008, the LVEDV of the ST36 group; *P* = 0.024, the LVESV of the PC6 group; *P* = 0.043, the LVESV of the ST36 group; *P* = 0.017, the LVEF of ST36 group after acupuncture). Data of LVEF, LVEDV, and LVESV of PC6 group were analyzed by *t*-test, and LVESV of ST36 group was analyzed by Wilcoxon signed-ranks. ^△^*P* < 0.05, compared with that of the PC6 group after acupuncture. The left ventricular wall mass of the ST36 group was significantly greater than that of the PC6 group (*P* = 0.030). Data were analyzed by *t*-test.
